# Evidence for the Dimerization-Mediated Catalysis of Methionine Sulfoxide Reductase A from *Clostridium oremlandii*

**DOI:** 10.1371/journal.pone.0131523

**Published:** 2015-06-24

**Authors:** Eun Hye Lee, Kitaik Lee, Geun-Hee Kwak, Yeon Seung Park, Kong-Joo Lee, Kwang Yeon Hwang, Hwa-Young Kim

**Affiliations:** 1 Division of Biotechnology, College of Life Sciences and Biotechnology, Korea University, Seoul 136–701, Republic of Korea; 2 Department of Biochemistry and Molecular Biology, Yeungnam University College of Medicine, Daegu 705–717, Republic of Korea; 3 Graduate School of Pharmaceutical Sciences, College of Pharmacy, Ewha Womans University, Seoul 120–750, Republic of Korea; University of Alberta, CANADA

## Abstract

*Clostridium oremlandii* MsrA (*Co*MsrA) is a natively selenocysteine-containing methionine-S-sulfoxide reductase and classified into a 1-Cys type MsrA. *Co*MsrA exists as a monomer in solution. Herein, we report evidence that *Co*MsrA can undergo homodimerization during catalysis. The monomeric *Co*MsrA dimerizes in the presence of its substrate methionine sulfoxide via an intermolecular disulfide bond between catalytic Cys16 residues. The dimeric *Co*MsrA is resolved by the reductant glutaredoxin, suggesting the relevance of dimerization in catalysis. The dimerization reaction occurs in a concentration- and time-dependent manner. In addition, the occurrence of homodimer formation in the native selenoprotein *Co*MsrA is confirmed. We also determine the crystal structure of the dimeric *Co*MsrA, having the dimer interface around the two catalytic Cys16 residues. A central cone-shaped hole is present in the surface model of dimeric structure, and the two Cys16 residues constitute the base of the hole. Collectively, our biochemical and structural analyses suggest a novel dimerization-mediated mechanism for *Co*MsrA catalysis that is additionally involved in *Co*MsrA regeneration by glutaredoxin.

## Introduction

**Fig 1:**
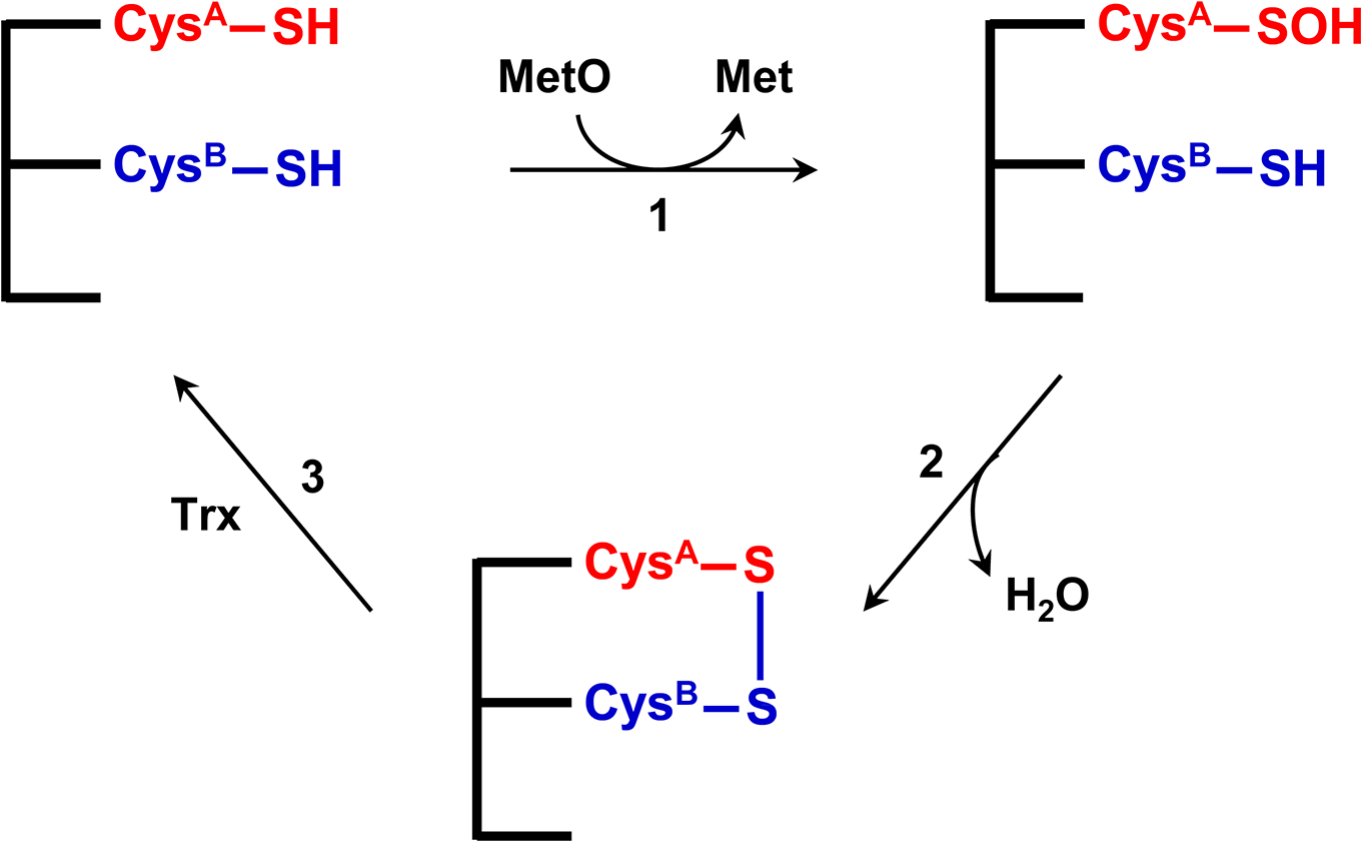
General catalytic mechanism of MsrA. The catalytic Cys^A^ (shown in red) attacks the sulfoxide of the substrate (MetO) to form a sulfenic acid intermediate, with concomitant release of Met (step 1). The resolving Cys^B^ (shown in blue) then reacts with the sulfenic acid intermediate on Cys^A^ to form an intramolecular disulfide bond (step 2), and the disulfide bond between Cys^A^ and Cys^B^ is further reduced by thioredoxin (Trx) (step 3). DTT can be used as the *in vitro* reductant.

Methionine sulfoxide reductase A (MsrA) is an important protein repair enzyme that specifically reduces methionine-*S*-sulfoxide to methionine. The general catalytic mechanism of MsrA comprises three steps that involve a common sulfenic acid chemistry (Fig 1) [1, 2]. A catalytic Cys attacks the sulfur atom of methionine sulfoxide to form Cys sulfenic acid, with the concomitant release of methionine. The catalytic Cys sulfenic acid then interacts with a resolving Cys, leading to the formation of an intramolecular disulfide bond. The enzyme is re-activated upon the reduction of the disulfide bond by reducing agents. The general *in vivo* reductant of MsrA is thought to be thioredoxin (Trx), while dithiothreitol (DTT) is effective *in vitro*.

The MsrAs fall into three groups based on the number of resolving Cys residues with which they are involved. 3-Cys MsrAs, such as those present in *Escherichia coli* and *Bos taurus*, contain two resolving Cys residues [3, 4]. A thiol-disulfide exchange reaction occurs between the two resolving Cys residues [5] and the resulting disulfide bond is reduced by Trx. 2-Cys MsrAs, such as those from *Mycobacterium tuberculosis*, have a single resolving Cys [6], while 1-Cys MsrAs, such as *Synechocystis* MsrA, lack resolving Cys residues [7]. The catalytic mechanisms for 3-Cys and 2-Cys MsrAs are well established [3–6, 8–11], but those for 1-Cys MsrAs have poorly been understood. The general catalytic mechanism, which involves the formation of an intramolecular disulfide bond between catalytic and resolving Cys residues, cannot be applied to 1-Cys MsrAs as they have no resolving Cys residue.

*Clostridium oremlandii* (strain OhILAs) is a strictly anaerobic, mesophilic, gram-positive bacterium, particularly rich in selenoproteins [12]. MsrA is present as a selenoprotein form in this organism [12]. The *Clostridium* MsrA (*Co*MsrA), consisting of 209 residues, is classified into a 1-Cys type MsrA as it has a selenocysteine (Sec) residue at the catalytic site but contains no Cys residues. The selenoprotein MsrA form has a 20-fold higher activity than its Sec-to-Cys form, showing that Sec is critical for catalysis [12]. However, the Sec-to-Cys version also shows activity comparable to other Cys-containing MsrAs [12, 13]. Interestingly, the *Co*MsrA is not reducible by Trx, the general *in vivo* reductant for MsrA [12], but efficiently reduced by glutaredoxin (Grx) [13, 14]. The *Co*MsrA is a monomeric protein in solution [15]. We previously determined the crystal structures of the 1-Cys type *Co*MsrA, including Sec-to-Cys form and its variants [15]. The *Co*MsrA structure folds into a common catalytic domain (residues 1–144) and a distinct helical domain (residues 145–209) which is absent from other known MsrA structures. We recently suggested a mechanism of *Co*MsrA regeneration by Grx, in which the catalytic Cys sulfenic acid intermediate is attacked by Grx to form a *Co*MsrA–Grx complex [16]. In this work, we report evidence that the *Co*MsrA enzyme can additionally undergo homodimerization during catalysis via a disulfide bond between catalytic Cys16 residues. We also describe a crystal structure of dimeric *Co*MsrA form, having the dimer interface around the two catalytic Cys16 residues.

## Materials and Methods

### Protein purification

Native selenoprotein *Co*MsrA was purified from *E*. *coli* BL21(DE3) as described previously [12]. The Sec-to-Cys form and its variants (E55A and E55D) were purified as described previously [15]. The *Clostridium* Grx1 mutant (U13C/C16S) was generated by site-directed mutagenesis using the construct pET-CLOS-Grx1/U13C as a template [14]. The monothiol form of Grx1 (U13C/C16S) was purified from *E*. *coli* BL21(DE3) by the same procedure with the *Co*MsrA.

### Dimerization assay

Sec-to-Cys *Co*MsrA (84 μM) was incubated with 0.2 mM free or dabsylated methionine sulfoxide for 2 h at 37°C in a buffer of 10 mM Tris-HCl (pH 8.0) and 100 mM NaCl. Other variants were similarly treated. The proteins in the reaction mixture were separated by non-reducing sodium dodecyl sulfate-polyacrylamide gel electrophoresis (SDS-PAGE). In the case of the native selenoprotein *Co*MsrA, 42 nM of protein was incubated under the same condition and then subjected to Western blot analysis. The kinetics of dimerization was analyzed using Prism 5 (GraphPad) software.

### Purification of dimeric *Co*MsrA

Purified monomeric *Co*MsrA was treated with 0.2 mM free methionine sulfoxide for 2 h at 37°C to induce its dimerization, and the reaction mixture passed through a Q ion exchange column to separate dimers from the remaining monomers. The eluted protein was concentrated into a volume of 2 ml and separated by size exclusion chromatography on a Superdex 200 column in 10 mM Tris-HCl (pH 8.0) and 100 mM NaCl. The >95% purity of the *Co*MsrA dimer was confirmed by non-reducing SDS-PAGE and the protein was concentrated to 10 mg/ml for crystallization.

### Reaction of dimeric *Co*MsrA with Grx

Purified dimeric *Co*MsrA (0.1 mM) was incubated with 0.1 mM monothiol form of Grx1 (U13C/C16S) for 2 h at 37°C in the buffer of 10 mM Tris-HCl (pH 8.0) and 100 mM NaCl. The reaction mixture was subjected to non-reducing SDS-PAGE.

### Mass spectrometry analysis

To identify the presence of a disulfide bond or a thiosulfinate bond between catalytic Cys16 residues of *Co*MsrA dimer, peptide sequencings were performed by nanoAcquity UPLC/ESI/MS (SYNAPT HDMS, Waters) combining with DBond algorithm [17]. Dimeric *Co*MsrA proteins separated by non-reducing SDS-PAGE were in-gel digested with trypsin followed by chymotrypsin, and the resulting peptides were extracted as previously described [18]. Concentrated peptides were separated using a C18 reversed-phase 75 μm i.d. × 250 mm analytical column (1.7 mm particle size, BEH130 C18, Waters) with an integrated electrospray ionization PicoTip (± 10 µm, New Objective). The MS/MS spectra were processed using the Micromass ProteinLynx Global Server (PLGSTM) 2.3 data processing software and output was collected as a single peak list (.pkl) file. The peak list files were applied to query the SwissProt database using Mascot (global search engine) and DBond (Korea, http://prix.hanyang.ac.kr/) [17], with the following parameters: peptide mass tolerance, 0.2 Da; MS/MS ion mass tolerance, 0.2 Da. Redundant peptides were excluded in the next run analysis using SEMSA to increase sequence coverage of each protein [19]. Reported assignments were verified by manual interpretation of spectra from Mascot and DBond.

### Crystallization and data collection

Crystallization of the purified *Co*MsrA dimer was initially performed with a commercial screen (Hampton Research) using the sitting drop vapor diffusion method at 20°C. After iterative optimization, a suitable crystal for data collection was obtained by the sitting drop vapor diffusion method using 0.1 M Tris-HCl, 1.2 M ammonium sulfate, and 12% (v/v) glycerol (pH 7.3). The crystal was exposed to a stream of liquid nitrogen at 100 K and subjected to X-ray diffraction. A 2.9-Å resolution native data set was collected on beamline BL1A at Photon Factory (Tsukuba, Japan) at a wavelength of 1.0000Å, and processed using the HKL2000 package [20]. The crystal belonged to the tetragonal space group P4_1_2_1_2 with a unit cell of dimensions a = b = 102.5 Å, c = 227.7 Å, and contained three molecules in an asymmetric unit. A summary of data collection statistics is provided in Table 1.

**Table 1:** Statistics on crystallographic analyses.

**Diffraction data**	
Space group	P4_1_2_1_2
Asymmetric unit	three molecules
Cell parameters (Å)	a = b = 102.5, c = 227.7
Resolution (Å)	2.90 (2.95−2.90)
*R*_sym_	0.122 (0.376)
*I/σ I*	11.6 (1.8)
Completeness (%)	94.6 (88.6)
Total reflections	139,543
Unique reflections	26,615
**Refinement**	
*R*_cryst_ (%)	24.8
*R*_free_ (%)	27.9
No. of atoms	
Protein	4,887
Ligand/ion	15
Water	48
B-factors	
Protein	78.1
Ligand/ion	99.4
Water	54.8
R.m.s deviations	
Bond lengths (Å)	0.004
Bond angles (°)	0.867
Ramachandran plot (%)	
Most favored	92.1
Allowed	7.1
Disallowed	0.8

The values in parentheses refer to the highest resolution shell.

### Crystal structure determination and refinement

The structure of the *Co*MsrA dimer was determined by molecular replacement based on the *Co*MsrA monomer structure (PDB code 4LWJ) as a search model using the software package PHASER implemented in PHENIX [21]. Further model building was performed using the Coot program [22] and refinement was executed with PHENIX [21]. The final model was validated using PROCHECK [23], and had an *R*_cryst_ of 24.8% and an *R*_free_ of 27.9%. A summary of statistics related to structure refinement is provided in Table 1. Protein Data Bank coordinates for dimeric *Co*MsrA have been deposited under the accession code 4U66.

## Results and Discussion

### *Co*MsrA can dimerize in the presence of substrate

**Fig 2:**
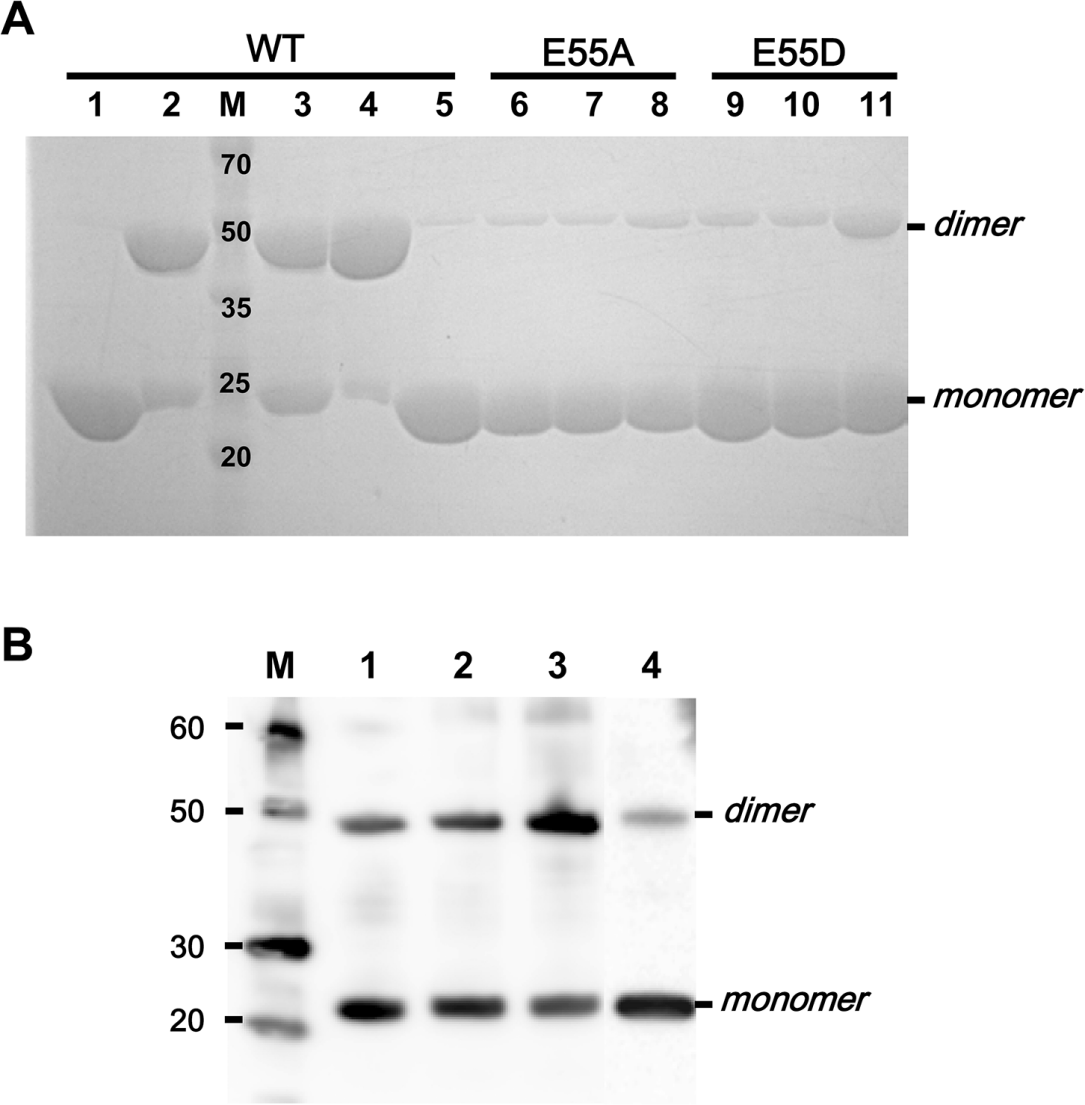
Dimerization of *Co*MsrA in the presence of substrate. (A) Dimerization of Sec-to-Cys *Co*MsrA and its variants. The dimerization was analyzed by non-reducing SDS-PAGE. A concentration of 0.2 mM free methionine sulfoxide (fMetO) or dabsyl-methionine sulfoxide (dMetO) was used. Lane 1, purified monomeric MsrA; lane 2, monomeric MsrA with fMetO; lane 3, monomeric MsrA with dMetO; lane 4, purified dimeric MsrA; lane 5, dimeric MsrA with 10 mM DTT; lane 6, purified E55A; lane 7, E55A with fMetO; lane 8, E55A with dMetO; lane 9, purified E55D; lane 10, E55D with fMetO; lane 11, E55D with dMetO. (B) Dimerization of native selenoprotein *Co*MsrA**.** The dimerization of natural Sec-containing MsrA was analyzed by non-reducing SDS-PAGE followed by Western blotting. Lane 1, purified selenoprotein MsrA; lane 2, selenoprotein MsrA with fMetO; lane 3, selenoprotein MsrA with dMetO; lane 4, the sample of lane 3 treated with 10 mM DTT.

*Co*MsrA is present in a monomeric state in solution and in crystal [15]. However, when *Co*MsrA protein is incubated with methionine sulfoxide and monothiol Grx, a minor band for homodimeric *Co*MsrA form is detected on a non-reducing gel, in addition to major bands for monomeric *Co*MsrA form and *Co*MsrA–Grx complex [16]. This observation led us to question whether dimerization of *Co*MsrA is additionally involved in its catalysis. *Co*MsrA protein was incubated at a concentration of 84 μM with the substrate for 2 h at 37°C. The *Co*MsrA mostly dimerized in the presence of both free and dabsylated methionine sulfoxide (Fig 2A, lanes 2 and 3). The dimer was even undissociated in the purification process (lane 4) but reduced to its monomeric form by the addition of DTT (lane 5). Since the catalytic Cys16 is the sole Cys residue in the protein, the data indicate that the formation of dimeric *Co*MsrA is likely via an intermolecular disulfide bond between the catalytic Cys16 residues. We attempted to analyze the intermolecular bond species by combining tandem mass spectrometry with DBond algorithm [17]. Disulfide bond formation between the two catalytic Cys16 residues from tryptic and chymotryptic peptides ^12^FALGCF^17^ and ^12^FALGCFWGPDAQ^24^ was detected, whereas no peptide containing a thiosulfinate bond was detected. However, the possibility is not absolutely excluded that the dimer could be formed with the thiosulfinate bond by condensation of the sulfenic acid molecules.

We tested whether dimerization occurs in the native selenoprotein *Co*MsrA during catalysis. Selenoprotein form of *Co*MsrA was incubated at a concentration of 42 nM with the substrate and the dimerization was analyzed by Western blotting (Fig 2B). Purified selenoprotein *Co*MsrA consisted of a mixture of dimers and monomers, even without exposure to the substrate methionine sulfoxide (lane 1). Addition of free methionine sulfoxide seemed to enhance dimer formation (lane 2). The dimer formation was clearly increased in the presence of dabsylated methionine sulfoxide (lane 3). DTT treatment reduced the dimer to the monomeric form (lane 4). Thus, these data indicate that the native selenoprotein *Co*MsrA can also dimerize during catalysis likely by the formation of an intermolecular diselenide bond between the catalytic Sec residues.

### Dimerization occurs in a concentration- and time-dependent manner

**Fig 3:**
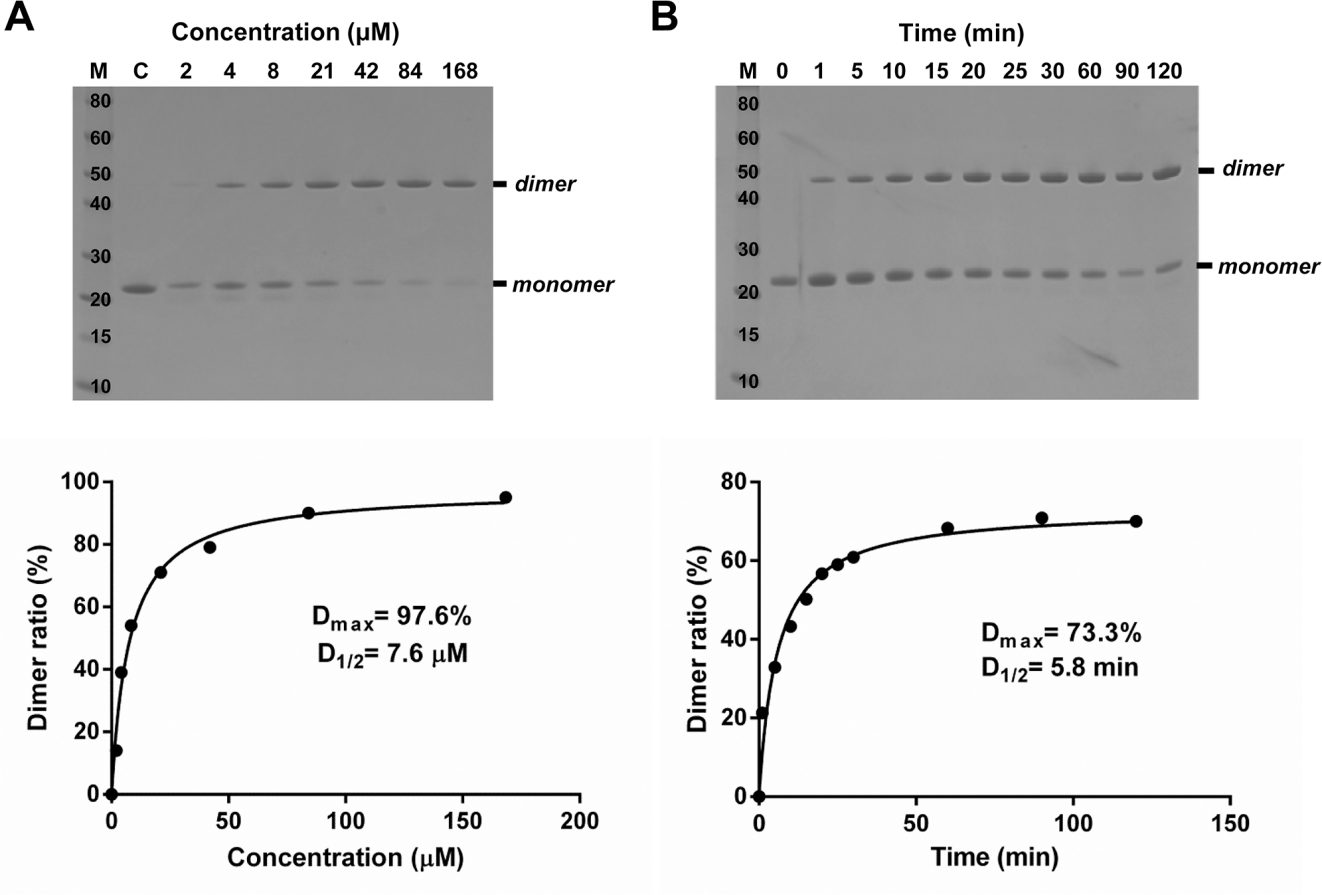
Kinetics of dimerization of *Co*MsrA. (A) Dimerization in response to the concentration of MsrA protein. The assay was performed for 2 h in the presence of 0.2 mM free methionine sulfoxide. (B) Dimerization in response to the reaction time. The assay was performed in the presence of 0.2 mM free methionine sulfoxide and 84 μM MsrA protein. Equal quantities of protein were loaded onto a non-reducing SDS-PAGE gel (upper panels). The dimer ratios were analyzed using the ImageJ program (lower panels). D_max_, maximal percent of dimer; D_1/2_, concentration of MsrA or reaction time to reach a half D_max_ value.

The dimerization rate was enhanced with an increasing protein concentration of MsrA (Fig 3A). The maximal percentage of dimers (D_max_) reached up to 98% and the concentration of MsrA forming dimers in the half maximum (D_1/2_) was 7.6 μM. The dimerization also occurred in a time-dependent manner (Fig 3B). The dimeric form was rapidly generated, with the final half of the dimers formed within 5.8 min.

### Dimerization is relevant for catalysis

**Fig 4:**
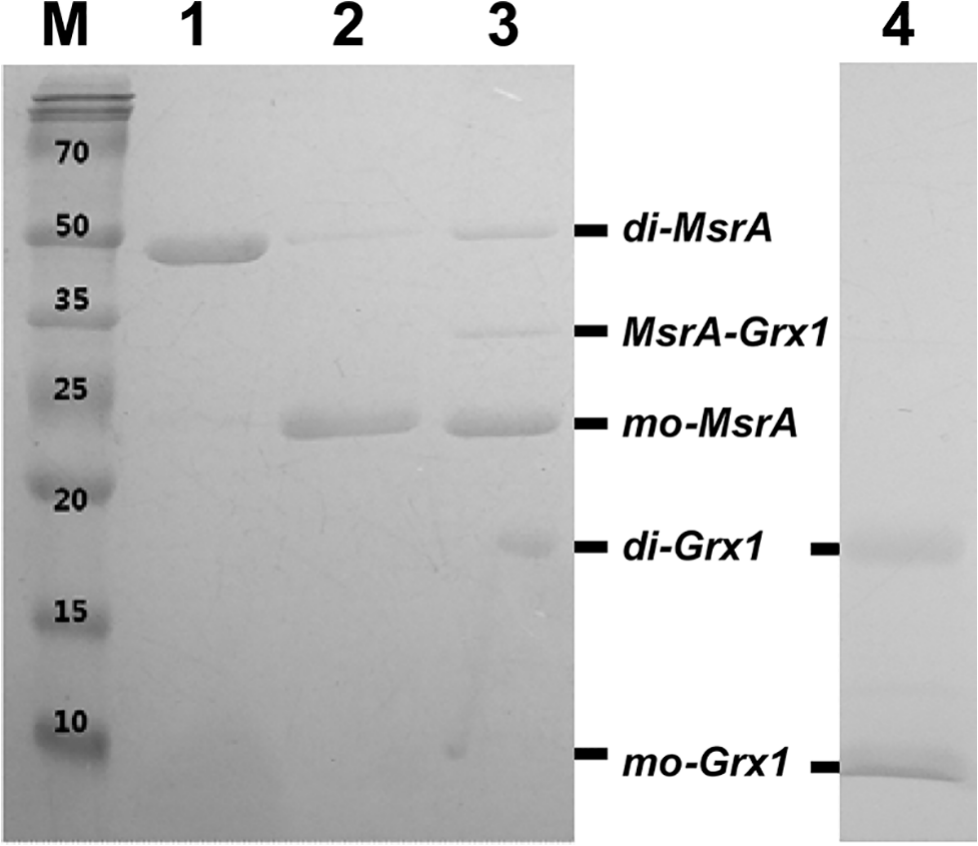
Resolution of dimeric *Co*MsrA by Grx analyzed by non-reducing SDS-PAGE. A monothiol form of Grx1 (U13C/C16S) is able to break the intermolecular disulfide bond of the MsrA dimer. Purified Grx1 is predominantly in a dimeric form (lane 4) and a Grx1-MsrA complex is formed by the reaction with dimeric MsrA (lane 3). Lane 1, dimeric MsrA; lane 2, dimeric MsrA treated with 100 mM DTT; lane 3, dimeric MsrA exposed to Grx1 (in the absence of glutathione); lane 4, Grx1 alone.

We confirmed whether the dimerization of *Co*MsrA is relevant for catalysis using almost completely inactive mutants, E55A and E55D. Glu55 acts as an essential residue for the *Co*MsrA catalysis by protonating and stabilizing the sulfoxide moiety. The E55A and E55D mutants show ~1000-fold lower activities than Sec-to-Cys wild-type [15]. Dimerization in the E55A variant was hardly detectable (Fig 2A, lanes 6–8). The E55D variant (lanes 9–11) was incapable of dimerization in the presence of free methionine sulfoxide (lane 10), but did form a small quantity of dimers when provided with dabsylated methionine sulfoxide (lane 11). In addition, the *Co*MsrA dimer could be resolved by the reductant Grx. As shown in Fig 4, a *Clostridium* Grx1 monothiol mutant (U13C/C16S) could cleave the intermolecular disulfide bond to form a mixed heterodimer. Taken together, our data demonstrate that the dimerization process is relevant for catalysis of *Co*MsrA.

### Dimeric structure of *Co*MsrA

**Fig 5:**
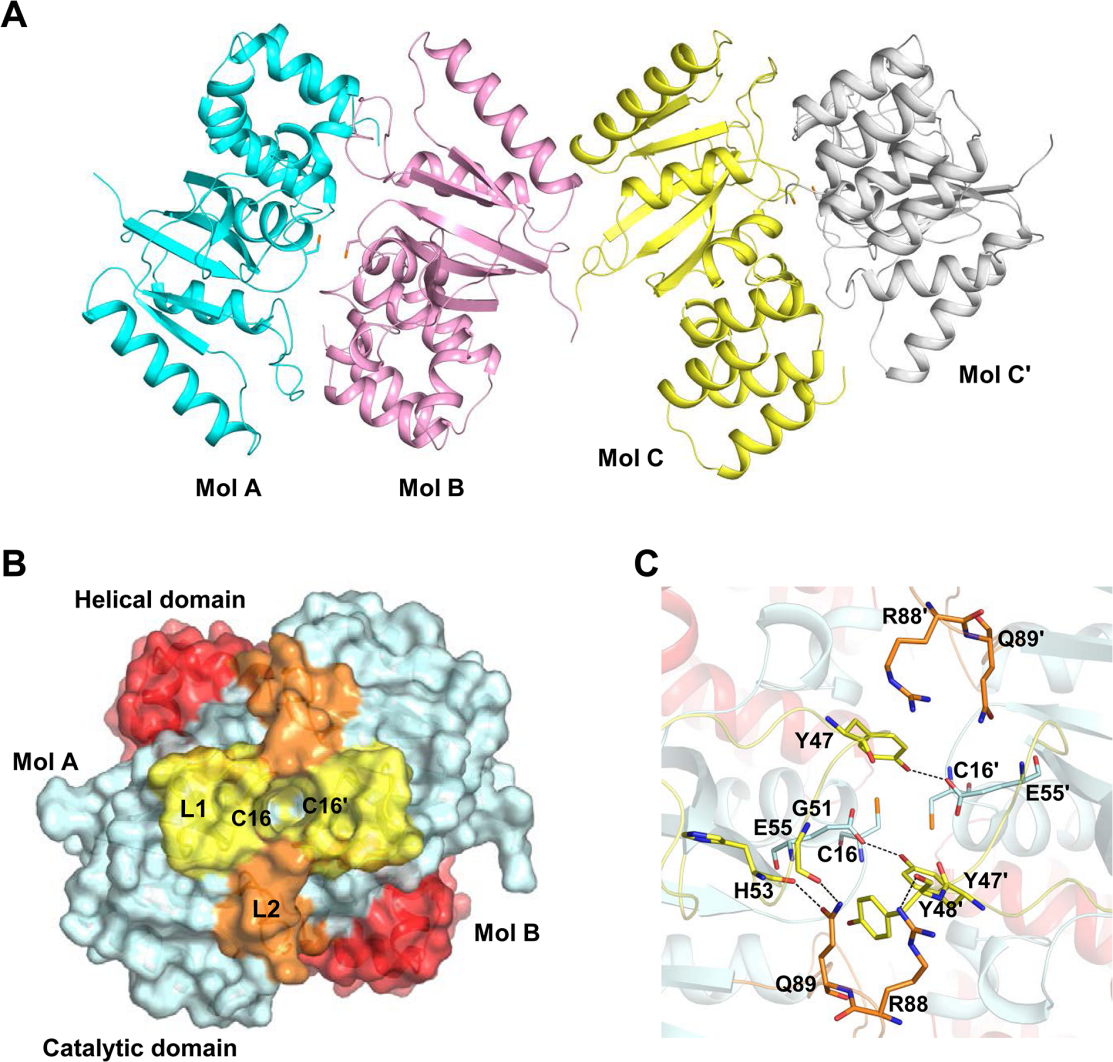
Dimeric structures of *Co*MsrA. (A) Overall structure of dimeric *Co*MsrA. Three molecules are included in the asymmetric unit. Molecules A (cyan) and B (pink) form a dimer, while molecule C (yellow) forms another dimer with its symmetric molecule C' (gray). The catalytic Cys16 residues are shown with stick models. (B) Surface model of homodimer structure formed by molecules A and B in the asymmetric unit. The loop regions L1 and L2 (yellow and orange) contribute to dimerization. The helical domain is shown in red. The dimeric MsrA, featuring the L1 and L2 loop regions, makes a hole at the center where the catalytic Cys16 residues are located. (C) The hole forming residues in the dimeric structure formed by molecules A and B. The residues are represented with stick models. The matching residues on the other monomer are indicated with the prime sign ('). The two residues on the L1 loop (Y47 and E55) and the two residues on L2 loop (Q89 and R88) play an important role in hole formation. Black dotted lines represent hydrogen bond interactions.

Dimeric *Co*MsrA was purified after substrate treatment to determine its crystal structure. The asymmetric unit contained one dimer and one monomer comprised of 204 residues (residues 5–208) (Fig 5A). The monomer in the asymmetric unit makes a dimer with a symmetric molecule. The superposition of the two dimeric structures shows an identical structure with a root mean square deviation (r.m.s.d.) of 0.26 Å for 389 Cα atom pairs. The area of the dimer interface was 1160 Å^2^ per monomer, corresponding to approximately 11% of the monomer’s surface area. The structure of monomeric *Co*MsrA consists of a catalytic domain (residues 1–144) and a helical domain (residues 145–209) [15]. This helical domain (absent in other MsrAs characterized so far) interacts with the catalytic domain and is essential for the active site formation [24]. The two catalytic domains from the pair of monomers face each other around the two catalytic Cys16 residues and occupy most of the dimer interface, whereas the helical domain lies distant from the interface (Fig 5B). Interestingly, a central cavity is present in the surface model of the dimer, with a diameter of ~9 Å and a depth of ~12 Å (Fig 5B). The cone-shaped hole has a maximum diameter of 9 Å and a minimum diameter of 6 Å. The two Cys16 residues constitute the base of the hole. The interior of the hole is mostly negatively charged, and the shape of the hole is symmetrical at the center of the dimer. A biochemical reason for the cavity formation in the dimeric MsrA is unclear.

The above dimerization assay indicated the joining of a homodimer by an intermolecular disulfide bond at Cys16 (Fig 2). However, although the two Cys16 residues were located close to each other in the dimeric MsrA structure within the asymmetric unit, no disulfide bond was observed (Fig 5C). The distance between the Cys residue sulfur atoms was 4.0 Å, which is far to make a viable disulfide bond (~2.05 Å). We checked the disulfide bond in the crystals. The dimer proteins from the dissolved crystals were mostly converted into monomeric forms by DTT treatment (S1 Fig). This result indicates that the intermolecular disulfide bond was present in the crystal before X-ray exposure. Thus, we speculate that the disulfide bond would be cleaved by intense synchrotron radiation [25, 26].

**Fig 6:**
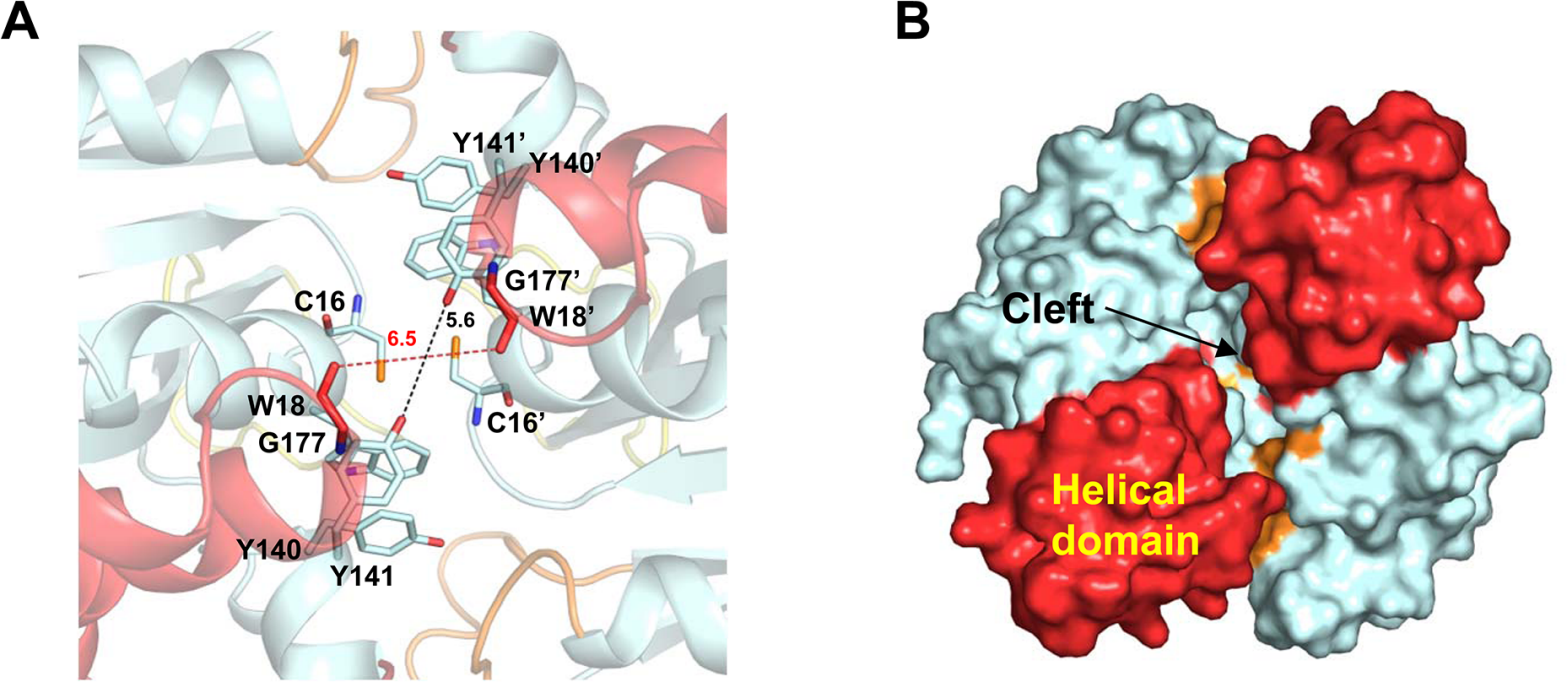
Rear view of dimeric *Co*MsrA. (A) The residues near the dimer interface of backside of dimeric MsrA are displayed with stick models. The distance between Y141 residues from each monomer is indicated with a black dashed line and the distance between G177 residues with a red one. (B) The surface model is represented, with the helical domain colored in red. Two helical domains are distantly located and make a crevice between two molecules.

In the dimer interface, the hydroxyl group of Tyr47 forms a hydrogen bond with the Oε1 atom of Glu55' at a distance of 2.6 Å (the “prime” sign indicates monomer B; Fig 5C). The residues ^48^YNLG^51^ (forming the L1 loop) define the entrance to the hole and the residue Gln89 on the L2 loop is positioned between Tyr48' and Gly51. The Oε2 atom of Gln89 interacts with the carbonyl oxygen of Gly51 (2.6 Å), and the Oε1 atom of Gln89 interacts with the carbonyl oxygen of His53 (2.5 Å). The Nη atom of the Arg88 residue interacts with the carbonyl oxygen of Tyr48' (2.8 Å). At the bottom side of the two Cys16 residues at its center, Trp18 is closely located (3.4 Å) at the backbone nitrogen of Cys16' and is stabilized by a π–π interaction with the aromatic Tyr140 side chain (Fig 6A). The aromatic side chain of Tyr141 is headed toward the dimer interface at a distance of 5.6 Å from that of Tyr141'. The two helical domains are located at the backside of the hole, contributing to the formation of a cleft, but no strong interactions are observed (Fig 6B). The closest distance between the two helical domains is 6.5 Å, which is formed between Gly177 residues located near the dimer interface. Since the two Cys16 residues are located at the bottom of the narrow cone-shaped hole, it is difficult for Grx to access the disulfide bond through this narrow hole. We assume that Grx protein can access the disulfide bond via the backside of the hole, which is a relatively open structure.

### Conformational changes of *Co*MsrA during catalysis

We previously determined monomeric structures of *Co*MsrA that represent structural redox states of the protein in reduced, substrate-bound, and sulfenic acid forms (PDB codes 4LWK, 4LWM, and 4LWJ, respectively) [15]. Overall, the three structures are highly similar, with an r.m.s.d. between 0.14 and 0.17 Å. However, there are conformational changes in the active site pockets of these structures [15]. The active site pocket forms an ‘open’ conformation in the reduced MsrA, which remains in the substrate-bound form. However, the ‘open’ active site pocket is changed to a ‘closed’ conformation in the sulfenic acid form. Interestingly, the active site pocket no longer exists in the dimeric MsrA with significant changes in the conformation of L1 and L2 loops, as described below. The Tyr47 residue on L1 loop is shifted towards the active site pocket. The residues Gln89 and Tyr90 on the L2 loop flip out and therefore contribute to dimer formation.

**Fig 7:**
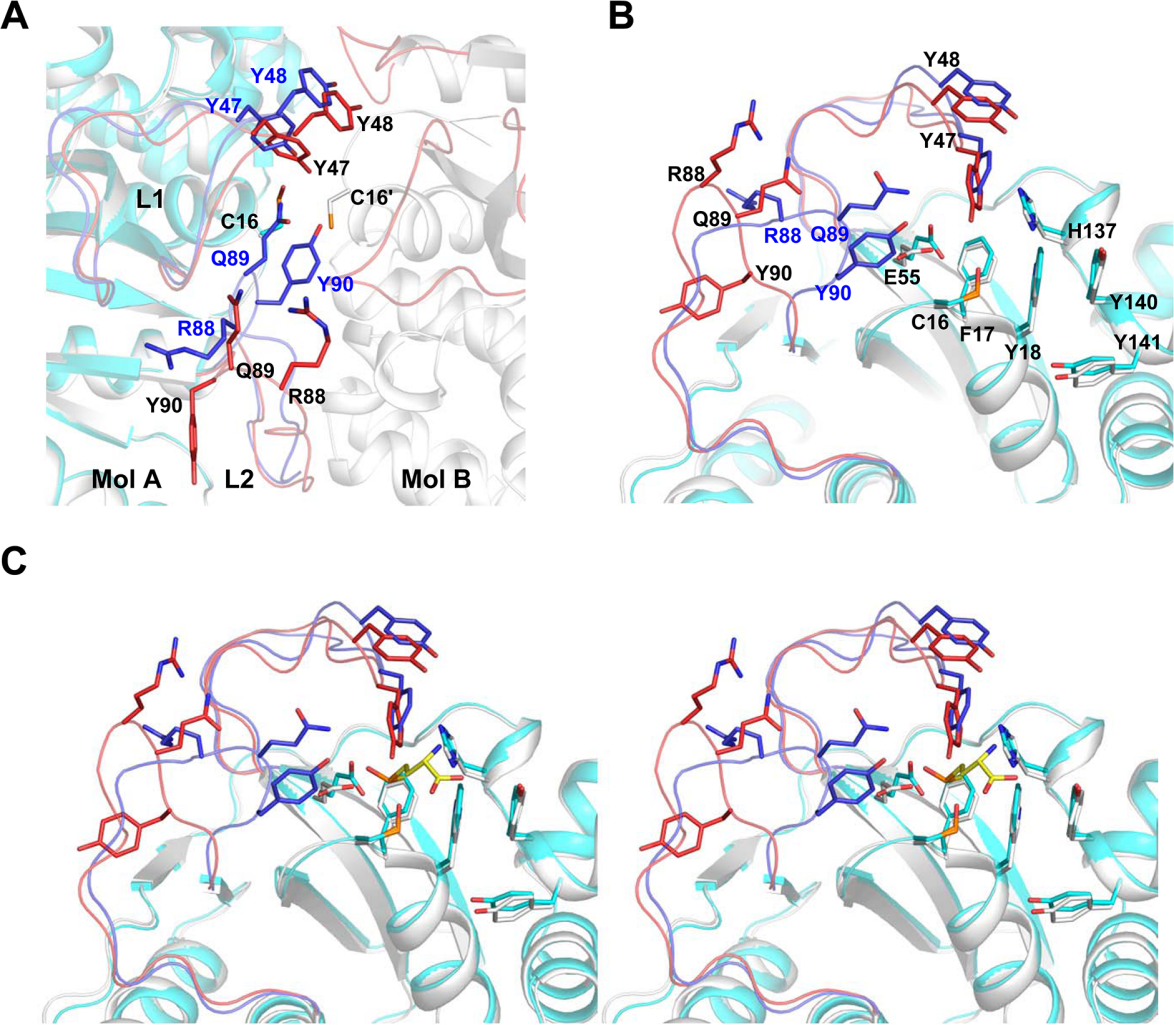
Conformational changes in dimeric *Co*MsrA structure. (A) Superposition of monomeric sulfenic acid form *Co*MsrA (PDB ID: 4LWJ, cyan) to a dimeric form (gray). L1 and L2 regions are shown in red (dimeric form) and blue (monomeric form). The two residues on L1 (Y47 and Y48) and three residues on L2 (R88, Q89 and Y90) are represented with stick models. (B) The active site residues and residues contributing to hole formation are indicated with stick models. The R88, Q89, and Y90 residues on L2 in the monomeric form are indicated in blue letters. (C) Stereo view of B. The substrate position (yellow stick model) was built in the active site using the substrate-bound structure (PDB ID: 4LWM).

The structure of dimeric MsrA was superimposed on the monomeric sulfenic acid form with an r.m.s.d. of 0.47 Å for 172 Cα atom pairs. This indicates a similar overall structure, except for the L1 and L2 loops (Fig 7A). Dimerization results in a shift of the L1 region towards the center of the dimer, while the L2 region must shift far from the center to offer sufficient space to fit another monomer. On the L1 loop itself, Tyr47 and Tyr48 are shifted by 1.6 Å and 2.2 Å, respectively, towards the center of the dimer; this allows them to form hydrogen bonds with the Oε1 atom of Glu55 and the backbone nitrogen of Gln89' (Fig 5C). The side chain of Glu55 is rotated by ~77°. This Glu55 residue acts as a proton donor to the sulfoxide oxygen during catalysis [15]. On the L2 loop, the ^86^TNRQYM^91^ stretch becomes displaced to provide space for the additional monomer (Fig 7B and 7C). Tyr90 is rotated by ~180° and shifted by 6.4 Å, providing the space to accommodate Tyr47'. The Gln89 side chain is shifted by 6.7 Å and is relocated between the two L1 loop regions. The active site residues Phe17, His137, and Tyr140 are somewhat rotated, while the positions of Cys16 and Trp18 are unaffected. Taken together, these conformational changes in the L1 and L2 loops are induced with the redox state changes from the sulfenic acid monomeric form to the dimeric form.

### A proposed model for dimerization-mediated catalysis of *Co*MsrA

**Fig 8:**
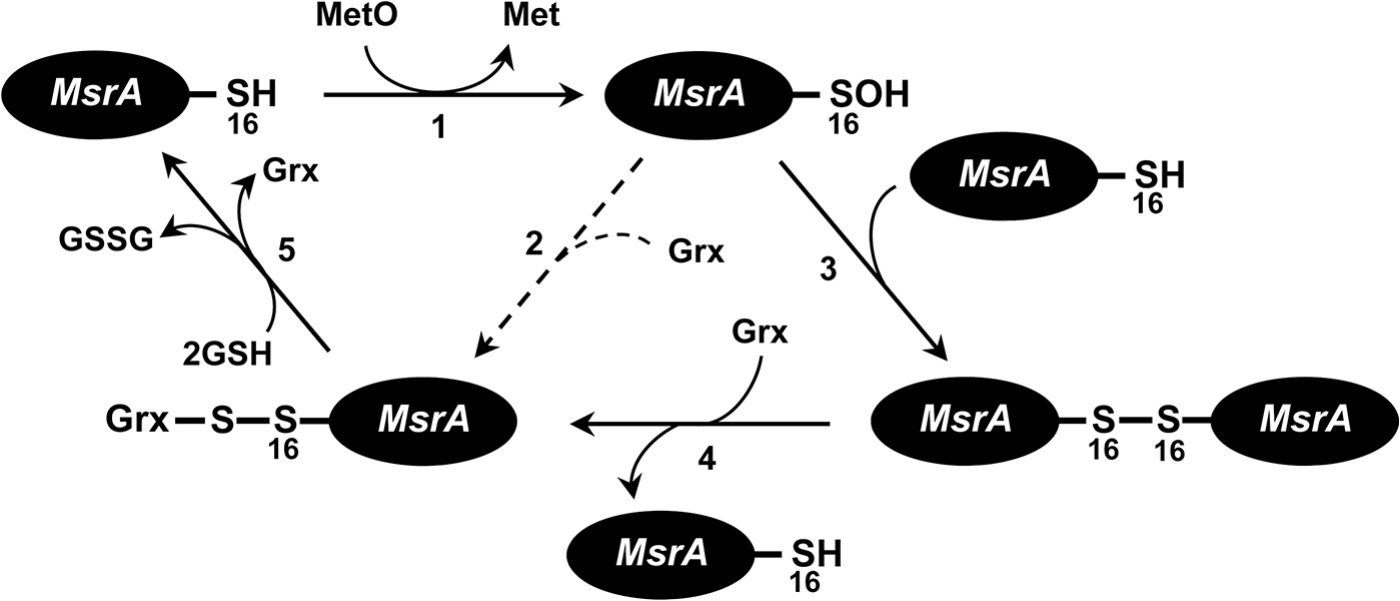
Proposed dimerization-mediated catalysis of *Co*MsrA. Catalytic Cys16 attacks the sulfoxide moiety of the substrate (MetO) and forms the sulfenic acid intermediate, with the concomitant release of Met (step 1). The Cys16 sulfenic acid is attacked by Grx as previously proposed [16] (step 2) or interacts with the catalytic Cys16 residue on a second *Co*MsrA molecule to form an intermolecular disulfide bond (step 3). The disulfide bond in the dimeric *Co*MsrA is attacked by Grx, leading to the formation of *Co*MsrA–Grx complex and the release of a reduced *Co*MsrA molecule (step 4). The mixed disulfide bond between monothiol Grx and *Co*MsrA is reduced by glutathione (GSH), leading to regeneration of another *Co*MsrA molecule (step 5). In the case of dithiol Grx, a thiol-disulfide exchange reaction may occur to resolve the mixed disulfide bond using the C-terminal resolving Cys of the active site of Grx.

Our biochemical and structural analyses suggest an additional dimerization-mediated catalysis of 1-Cys type *Co*MsrA via the formation of an intermolecular disulfide bond (Fig 8). The thiol group in the catalytic Cys16 attacks the sulfur atom of methionine sulfoxide to form sulfenic acid, with the concomitant release of Met. The catalytic Cys16 sulfenic acid is attacked by Grx to form a *Co*MsrA–Grx complex or additionally interacts with the catalytic Cys16 residue of the other monomer, leading to the formation of a dimer via a disulfide bond. The intermolecular disulfide bond is reduced by the reductant Grx. Dimerization may favorably occur in a high concentration of the *Co*MsrA enzyme.

## Conclusions

We report dimerization-mediated catalysis of 1-Cys type *Co*MsrA and its dimeric structure. The *Co*MsrA enzyme can dimerize after substrate reduction via an intermolecular disulfide bond between the catalytic Cys residues. This dimerization mechanism can be used as an additional mechanism of *Co*MsrA regeneration by Grx. The crystal structure of dimeric *Co*MsrA reveals insights into dimer interface, the role of interfacing residues in dimer formation, and conformational changes from the sulfenic acid monomeric form to the dimeric form.

## Supporting Information

S1 Fig The presence of disulfide bond in the dimeric *Co*MsrA crystals. The dissolved crystals were subjected to SDS-PAGE analysis. Lane 1, non-reduced; lane 2, reduced (10 mM DTT treatment).
(TIF)
