# Supplementary figures and images for: Evidence for the Dimerization-Mediated Catalysis of Methionine Sulfoxide Reductase A from Clostridium oremlandii
Source: PLoS One. 2015 Jun 24;10(6):e0131523. doi: 10.1371/journal.pone.0131523 (PMC4479559; doi:10.1371/journal.pone.0131523)

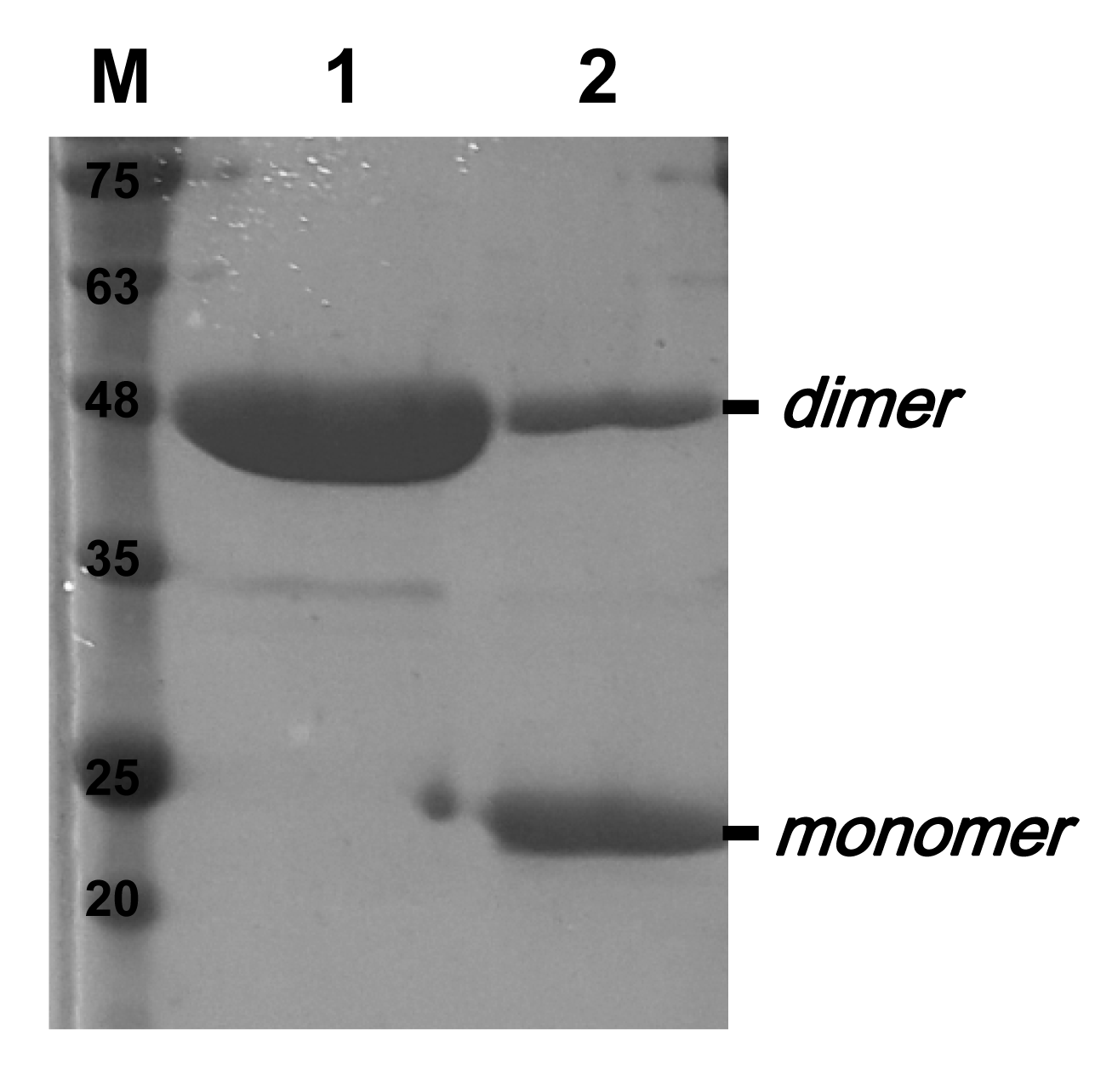

Supplement: S1 Fig — The dissolved crystals were subjected to SDS-PAGE analysis. Lane 1, non-reduced; lane 2, reduced (10 mM DTT treatment). (TIF) [file pone.0131523.s001.tif]
